# Integrated Performance Evaluation of Aerogel-Based Fibre-Enhanced Thermal Renders Applied on Building Walls

**DOI:** 10.3390/gels9110898

**Published:** 2023-11-13

**Authors:** Marco Pedroso, José Dinis Silvestre, Maria da Glória Gomes, Ahmed Hawreen, Jéssica D. Bersch, Inês Flores-Colen

**Affiliations:** 1 Civil Engineering Research and Innovation for Sustainability (CERIS), Departamento de Engenharia Civil, Arquitetura e Ambiente (DECivil), Instituto Superior Técnico (IST), Universidade de Lisboa, Av. Rovisco Pais, 1049-001 Lisbon, Portugal; marco.pedroso@tecnico.ulisboa.pt (M.P.); jose.silvestre@tecnico.ulisboa.pt (J.D.S.); maria.gloria.gomes@tecnico.ulisboa.pt (M.d.G.G.); hawreen.a@gmail.com (A.H.); jessica.d.bersch@tecnico.ulisboa.pt (J.D.B.); 2 Department of Highway and Bridge Engineering, Technical Engineering College, Erbil Polytechnic University, Erbil 44001, Iraq; 3 Department of Civil Engineering, College of Engineering, Nawroz University, Duhok 42001, Iraq; 4 Núcleo Orientado para a Inovação da Edificação (NORIE), Programa de Pós-Graduação em Engenharia Civil: Construção e Infraestrutura (PPGCI), Universidade Federal do Rio Grande do Sul (UFRGS), Av. Osvaldo Aranha, 99, Porto Alegre 90035-190, Brazil

**Keywords:** ADP-ff, aramid fibres, cost optimisation, GWP, optimum insulation thickness, payback period, silica aerogel, sisal fibres, thermal render

## Abstract

In this work, aerogel renders were enhanced with fibres for use in new building walls, emphasising a Mediterranean climate. The main novelty of the study relies on an integrated evaluation of the aerogel-based fibre-enhanced thermal renders from environmental, energy and economic approaches. Therefore, optimum insulation thicknesses, life cycle savings, payback periods, abiotic depletion potential from fossil fuels (ADP-ff) and global warming potential (GWP) impacts were quantified as a function of the energy consumption. The cost optimisation of aerogel-based renders enabled a reduction from 2477.4 to 1021.7 EUR∙m^−3^ for the reference formulation, and the sisal-optimised render led to the best-integrated performance. A higher DD* (degree-days equivalent) led to higher optimum thicknesses (the Azores required 0.02 m and 0.01 m and Bragança 0.06 m and 0.03 m for cost-optimised and non-optimised thermal renders with sisal fibre, respectively). The optimum thickness related to the ADP-ff and GWP impacts was higher, 0.04 m for the Azores and 0.09 m for Bragança. A steeper decrease in the annual energy consumption occurred for thermal renders up to 0.02 m in the Azores and 0.04 m in Bragança. Aerogel-based fibre-enhanced thermal renders had benefits, mainly from 600 DD* onwards.

## 1. Introduction

The concerns towards climate change have led to several mitigation plans, such as the objective of making the climate neutral by 2050 in the European Union, with a significant milestone on emission reduction by 2030. This is in agreement with the Paris Agreement [1], with the main action lines related to reducing non-renewable energy sources and emissions of greenhouse gases [2]. However, simultaneously, concerns referring to the indoor comfort of building’s users [3,4,5] have led to higher levels of heating and cooling energy consumption, which have made the European built environment responsible for approximately 40% of the total energy consumption and 36% of CO_2_ emissions [3,4]. Therefore, urgent solutions are needed to reduce the resulting impacts and achieve climate-neutral objectives.

One of the best-known ways to spare energy in buildings is by applying thermal insulation on their envelope [5,6,7,8], which has led to the development of innovative materials. Among them, nanomaterials (such as silica aerogel) have been used as thermal insulating aggregates [9,10,11,12,13,14], with research on their applications and formulations [15,16,17]. The interest in using such an aggregate is due to its low thermal conductivity (below 0.021 W∙m^−1^∙K^−1^ [18]), resulting from its nanoporous structure and highly tortuous paths that limit heat transport by radiation, convection and conduction [19]. Therefore, using aerogels in the building sector may contribute to energy conservation and emission reduction [20].

However, some limitations can impair the application of aerogel-based thermal renders on building envelopes, such as their low mechanical strength [21] and high capillary water absorption [9,22,23]. Two main effective methods have been followed to overcome such limitations: the introduction of fibres [24,25,26] and the application of a multilayered protective coating system on the renders’ surface, which is composed of a basecoat, a fibreglass mesh, a key coat and a finishing coat [27], similar to the ones applied over ETICS (external thermal insulation composite systems) [28]. Hygrothermal [29] and environmental [30] characterisations verified that aerogel-based fibre-enhanced thermal renders show multifunctionality.

Although some integrated approaches already exist regarding the environmental, energy and economic savings of thermal insulation [31,32,33], the most common method of selection and determination of the optimum thicknesses is based on degree-day heat loss analysis [34]. This method considers the initial cost of the thermal insulation material and the energy savings throughout the entire service life, allowing for the determination of the optimum insulation thickness that corresponds to the minimum service life cost [35]. Simultaneously, a similar approach provides the thickness that minimises the total life cycle environmental impact [36].

Many studies have determined the optimum insulation thicknesses of conventional thermal insulating materials on building walls considering economic and energy aspects. However, when environmental impacts are considered, the number of available studies significantly decreases. Hasan [37] studied the application of expanded polystyrene (EPS) and rock wool thermal insulation boards over building assemblies in Palestine, considering different heating fuels. Similar studies for other locations were conducted by Çomakli and Yüksel [38] and Sisman et al. [39]. Ozel [40,41,42] evaluated several insulation materials, concluding that they present different optimum thicknesses when considering the economic impacts of energy consumption, the material costs and the environmental impact indicators such as carbon dioxide (CO_2_) and sulphur dioxide (SO_2_). A similar approach was presented by Sagbansua and Balo [43], Çomakli and Yuksel [44], Ucar and Balo [45] and Yu et al. [6] for other locations and applications. Dombayci et al. [46], Ucar and Balo [47] and Bolattürk [48] studied the influence of different energy sources and their economic and environmental impacts on the optimal thickness of insulating materials, verifying that electricity was one of the most cost-saving solutions compared with other energy sources, such as coal or fuel oil. Thus, factors that significantly influence the optimum insulation thicknesses, payback periods and energy savings include the location’s climatic characteristics, the energy sources, the constructive reference solution and the service life [46,47,48].

As for silica aerogel-related materials, the study of Cuce et al. [49,50] focused on filling cavity walls with them following a similar investigation as Ozel [40]. They analysed the optimum thicknesses based on the environmental impacts of CO_2_ and SO_2_ emissions. The same approach was followed by Huang et al. [51] for aerogel blankets applied in Chinese buildings. These studies showed that, depending on the application context, the higher initial impacts of any insulating material correspond to lower accumulated life cycle impacts regarding the optimisation of their thickness and considering the operational energy impacts over the service life.

Ibrahim et al. [11,52] followed a similar approach to previous research for aerogel-based renders but did not study environmental impacts. In Portugal, although Garrido et al. [53] investigated the life cycle cost of several aerogel-based thermal renders, they did not evaluate the associated environmental impacts or optimise the different solutions’ thicknesses. Furthermore, only recently, LCA data for silica aerogel became available [31,49,50,54,55]. Boccia et al. [56] summarised regulatory and safe handling procedures regarding nanostructured aerogels, aiming to reduce human and environmental risks, mainly due to the lack of data on the topic. A thorough study of the aerogel-based thermal renders was performed [30] regarding the anticipatory modelling [57,58]. However, the material potential for environmental, energy and economic savings in buildings [12,32] still has to be quantified, pointing to a lack of knowledge on comprehensive and quantified data regarding an integrated approach which includes comparisons with other existing thermal insulators.

In this context, the present study was designed and based on aerogel-based fibre-enhanced thermal renders previously characterised in terms of their physical, mechanical, microstructural, hygrothermal and environmental aspects [26,29,30]. The prepared mixes included a reference thermal render with no fibres, a thermal render with 0.5% (vol.) of aramid fibres, one with 0.1% (vol.) of sisal fibres and another with 0.1% (vol.) of biomass fibres. A sensitive analysis of their costs was carried out, and the application of the thermal render enhanced formulations with sisal and aramid fibres was simulated in a new wall on a Mediterranean climate regarding Portugal’s two most distinct climatic regions, the Azores and Bragança. Two economic scenarios were assessed for the thermal renders: a non-optimised scenario and an optimised scenario, considering the expected decreases in silica aerogel costs. Finally, other locations were considered based on their cooling and heating degree-days (CDD and HDD, respectively). The study’s main objective is to evaluate the aerogel-based fibre-enhanced thermal renders’ environmental, energy and economic performance, aiming to reinforce their application potential on building envelopes for improving energy efficiency while reducing the associated costs and environmental impacts.

## 2. Results and Discussion

**Figure 1:**
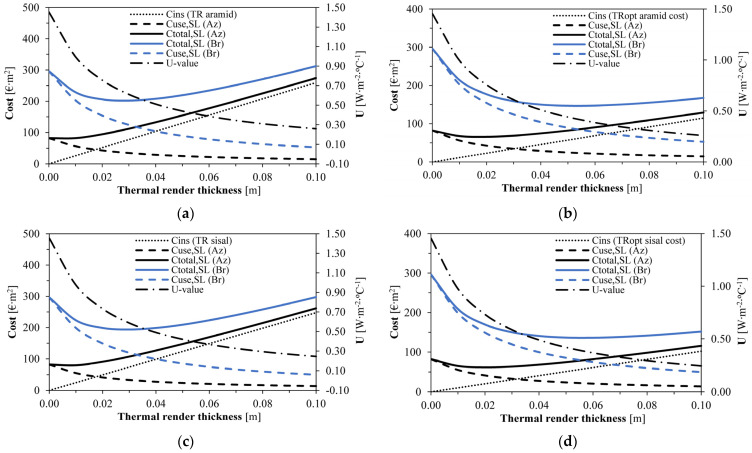
Optimum insulation thickness for the aramid and sisal thermal renders (Az—Azores; and Br—Bragança): (**a**) Aramid−based thermal render (TR aramid); (**b**) Aramid−based optimised costs thermal render (TRopt aramid); (**c**) Sisal−based thermal render (TR sisal); (**d**) Sisal−based optimised costs thermal render (TRopt sisal).

In Table 1, the main results of the simulations are presented. Figure 1 illustrates the cost comparison for the economically non-optimised (TR aramid and TR sisal) and optimised formulations (TRopt aramid and TRopt sisal). In all scenarios, the energy costs decreased with higher insulation thickness, and the insulation costs linearly increased, influencing the total cost. The optimum thickness can be graphically identified since the point with minimum total cost (thermal insulation cost plus energy cost) represents the most cost-effective application [59].

The Azores present a significantly different degree-days equivalent (DD*) compared to Bragança (188.8 vs. 680.1 °C∙day, Table 1), which impacts the optimum thermal insulation thickness, leading to a much thinner layer (0.01 for the Azores vs. 0.03 m for Bragança) regarding the non-optimised formulations. Therefore, the more demanding and colder climate of Bragança results in a higher optimum thermal insulation thickness [38,39,47] due to the higher energy needs for indoor climatisation, which dilutes the thermal insulating material costs [40].

In regions like the Azores, which are more temperate with a lower DD*, the energy needs are lower and the thermal insulation costs more significantly influence the optimum thickness by reducing it. This issue is especially evident when the non-optimised and optimised formulations are compared because the silica aerogel optimised cost allows for more economic acquisition of the renders, compensating for higher energy costs, with TRopt sisal showing the best performance.

As for the impact on the thermal transmittance of the walls (U), the requirements for the Azores and Bragança are U ≤ 0.45 W∙m^−2^∙°C^−1^ and U ≤ 0.35 W∙m^−2^∙°C^−1^, respectively [60]; thus, only the TRopt sisal nearly achieves it. If the required U-value was an initial concern in this assessment, a different constructive solution should have been considered, like a wider lightweight concrete block.

The payback period (desirably short [50,61]) of almost all formulations surpassed the service life of 50 years in the Azores, as shown in Table 1. On the other hand, for Bragança, which has higher energy needs, payback periods dropped significantly to less than 20 years due to insulation improvement, energy savings and related impacts.

Figure 1 shows that the insulation cost restricts the choice for a thicker layer in the non-optimised formulations, with lower impacts for the cost-optimised. Moreover, in the Azores, the optimised formulations improved the S_SL_ 8 to 10 times compared to the non-optimised. In Bragança, although only improving around two times, they represented an additional saving of ≈60 EUR∙m^−2^ during the 50 years.

**Figure 2:**
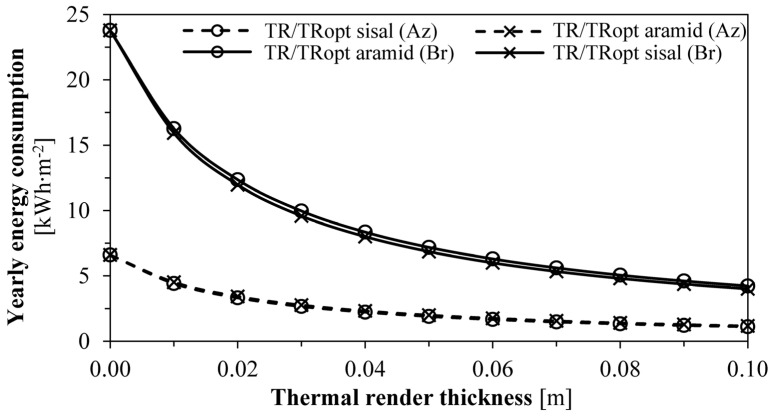
Annual energy consumption (Az—Azores; and Br—Bragança).

Regarding energy consumption, Figure 2 illustrates that higher insulation thicknesses increase energy savings. There is a steeper decrease in energy consumption up to a thickness of 0.02 m in the Azores and 0.04 m in Bragança, which represent absolute energy savings of ≈5 and 15 kWh∙m^−2^∙year^−1^, respectively, when compared with the uninsulated wall.

In Bragança, the thermal insulation thickness had a higher influence on the energy consumption reduction (Figure 2) due to its thermal conductivity. In this case, the economic optimisation has no direct influence since the thermal conductivities of the economically non-optimised and optimised formulations are the same. For example, the use of 0.01 m of thermal render, either aramid- or sisal-enhanced, can lead to an annual operational energy saving of 7.86 kWh∙m^−2^ in Bragança and of 2.10 kWh∙m^−2^ in the Azores due to their similar thermal conductivities.

**Figure 3:**
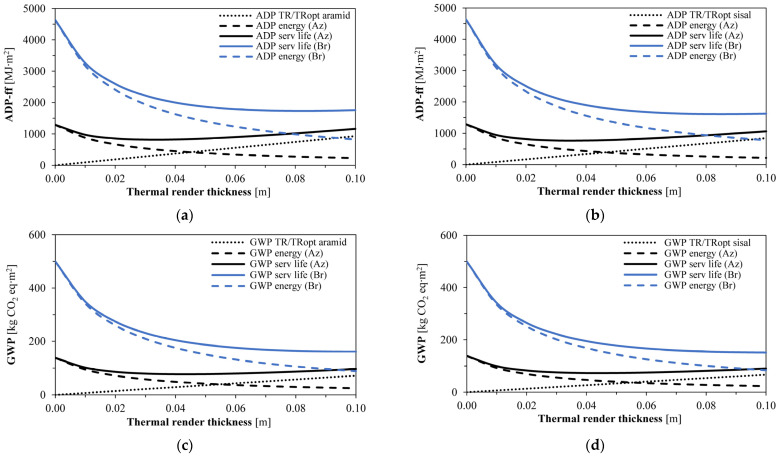
Environmental indicators for the different thicknesses of the aerogel−based fibre−enhanced thermal renders (Az—Azores; and Br—Bragança): (**a**) Aramid−based thermal renders ADP−ff (TR and TRopt aramid); (**b**) Sisal−based thermal renders ADP−ff (TR and TRopt sisal); (**c**) Aramid−based thermal renders GWP (TR and TRopt aramid); (**d**) Sisal−based thermal renders GWP (TR and TRopt sisal).

A similar trend for the ADP-ff and GWP environmental impacts is observed compared to the energy behaviour (Table 1 and Figure 3). As the insulation thickness increases, the energy consumption and environmental impacts decrease until an optimum point. The environmental impacts associated with the thermal renders were the same between economically non-optimised and optimised formulations.

In Bragança, there was a more significant decrease in the environmental impacts with the increase in the thermal insulating thicknesses; the higher DD* and optimum thicknesses (sparing more energy for heating and cooling) reduced the environmental payback periods. In the Azores, only slight improvements were observed compared to the reference uninsulated wall due to lower energy needs. Moreover, since the Azores present a lower DD*, the optimum thicknesses were also lower, leading to increased payback periods regarding the environmental indicators.

As depicted in Figure 3, an optimum thickness related to the ADP-ff and GWP impacts would increase to 0.04 m for the Azores and 0.09 m for Bragança compared to the economic analysis, as represented in the lowest points of the service life curves. With this selection, the material with the highest thermal conductivity (TR/TRopt aramid) presents a U of 0.51 W∙m^−2^∙°C^−1^, still above the legislation requirements of 0.45 W∙m^−2^∙°C^−1^ [60], but for Bragança, a U of 0.28 W∙m^−2^∙°C^−1^ is achieved, while 0.35 W∙m^−2^∙°C^−1^ are required [60]. This can lead to a discussion regarding the governmental funding of thermal insulating solutions and the expected reductions in the overall environmental impacts [62].

**Figure 4:**
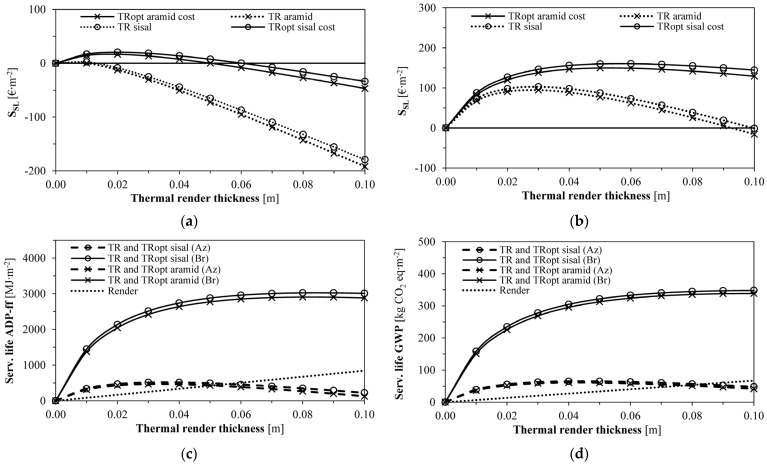
Service life economic and environmental impacts savings (Az—Azores; and Br—Bragança): (**a**) Service life economic savings—Azores; (**b**) Service life economic savings—Bragança; (**c**) Service life ADP−ff savings; (**d**) Service life GWP savings.

As expected, regarding the overall economic and environmental service life savings shown in Figure 4, the highest economic savings corresponded to the optimum thickness with the optimised formulations (TRopt). Due to the lower climatic variations, the Azores showed lower savings than Bragança (20.5 vs. 160.4 EUR∙m^−2^, for TRopt formulations). A 0.02 m thickness can save ≈21 EUR∙m^−2^ for the Azores, while a 0.04 m thickness can save ≈156 EUR∙m^−2^ for Bragança regarding the considered life cycle. Regarding the environmental indicators, the sisal-containing formulation (TR/TRopt sisal) resulted in slightly higher savings (≈5%) than aramid (TR/TRopt aramid), leading to a ≈40% improvement in the Azores and a ≈60% improvement in Bragança. Even for small insulation thicknesses (e.g., 0.02 m in the Azores and 0.04 m in Bragança), the environmental savings were significant, as also verified by other research for conventional thermal insulating materials [43,44,45].

Significant environmental, energy and economic savings were obtained when the solutions were compared with the corresponding uninsulated wall. Even for the less demanding climatic region, the Azores, savings were obtained in all indicators while also increasing indoor comfort, as seen through lower U-values, leading to a reduction in the cold wall effects [63].

**Figure 5:**
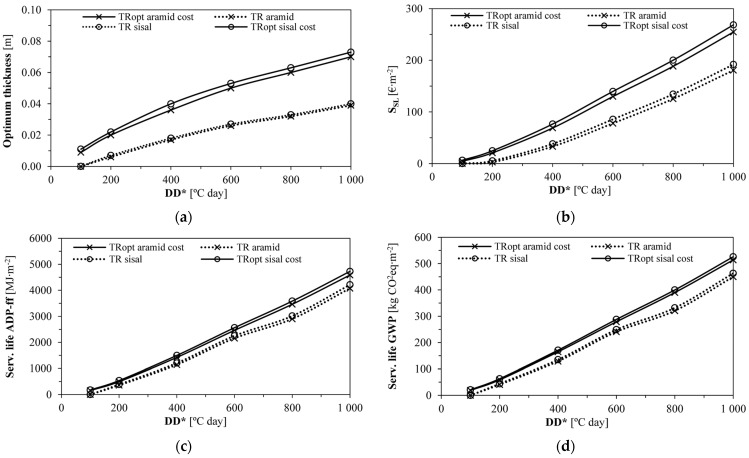
Influence of the DD* on the optimum thickness and respective impacts (economic and environmental): (**a**) Optimum thickness as a function of DD*; (**b**) Service life economic savings for the optimum thickness; (**c**) Service life ADP−ff savings for the optimum thickness; (**d**) Service life GWP savings for the optimum thickness.

Figure 5 allows the evaluation of different DD* scenarios, covering all regions of Portugal. As expected, increases in the degree-days enabled higher optimum thicknesses; the economically optimised formulations led to higher optimum thicknesses due to the lower insulation cost. Thus, the optimised formulations led to economic and environmental savings, more accentuated from 600 DD* onwards, since they reduced the energy needed to regulate the indoor climate due to higher thicknesses and lower U-values. These results could be extrapolated for other regions in Europe, colder than Bragança [64], where the studied thermal renders could also reduce energy consumption and environmental impacts.

The curves in Figure 5c,d are related to optimum thicknesses (Figure 5a). The economically non-optimised formulations are around 0.03 m, which lowers their environmental contribution. TRopt sisal showed the best performance for all the indicators. Thus, more demanding climates need better-performing materials since the energy savings can be much higher, reducing the arising emissions and costs. The optimum thicknesses, payback periods and energy and environmental impact savings are closely related to the thermal conductivity. For the same thickness, a material with lower thermal conductivity increases the thermal insulation, reducing the energy needs [40,43,65].

If more characteristics of the innovative aerogel-based fibre-enhanced thermal renders were considered in the study, as their hygrothermal behaviour (e.g., water vapour permeability—[29]), the results could be even more attractive [66,67]. Karim et al. [68], for instance, studied the moisture absorption behaviour of aerogel-based coatings when exposed to different wetting scenarios.

Regarding the assumptions and limitations of the simulations, the selected geographical locations and orientations may have influenced the results [69]. Furthermore, it was considered that 100% of the time—an occupation of the construction 24 h per day—the temperature limits (between 18 and 25 °C) were accomplished. Also, the PWF (inflation and interest rates), indoor climate temperature set-points, efficiency ratios, energy costs, environmental impacts and service life periods can impact the results [46,59,70].

Lastly, the energy source used and its impacts and costs were kept constant throughout the service life. As seen in other studies [40,47,50], the impacts and costs associated with the energy source can increase over time. In these cases, the thermal insulation materials’ optimum thicknesses can also increase since their direct costs and impacts become more diluted in those referring to the energy sources.

## 3. Conclusions

This study presents the calculation of the optimum insulation thicknesses of aerogel-based fibre-enhanced thermal renders when applied on building facades under distinct climatic (DD*) locations in Portugal: the Azores and Bragança. Aramid and sisal fibres were selected for investigation.

Firstly, a sensitivity analysis was carried out concerning the aerogel cost. Then, a new wall applied with the thermal insulation was analysed regarding cost-optimised and non-optimised scenarios. The research assessed three main perspectives: environmental, energy and economic, through the evaluation of global cost savings, energy savings, payback periods and environmental impact reductions—abiotic depletion from fossil fuels (ADP-ff) and global warming potential (GWP)—of the thermal insulation solutions compared to the uninsulated wall.

With the results obtained, it is possible to draw the following main conclusions:

- The optimised aerogel-based renders (TRopt) showed a better performance, as expected since their acquisition costs were significantly lower (from 2477.4 to 1021.7 EUR∙m^−3^ for TR reference). Based on the latest findings, it is believed that this cost reduction will be possible. The sisal-enhanced formulation (TRopt sisal) showed the best integrated performance of all the considered thermal renders;
- The Portuguese region with the highest DD* (degree-days equivalent) led to higher optimum thicknesses (e.g., the Azores with 0.02 m and 0.01 m and Bragança with 0.06 m and 0.03 m for cost-optimised and non-optimised thermal renders with sisal fibre, respectively), since the use of more energy for indoor temperature regulation diluted the impacts of the thermal render;
- In the Azores, the cost-optimised formulations improved the savings during the solutions’ service life (S_SL_) from 8 to 10 times compared to the non-optimised, while in Bragança, although the improvement was around two-fold, it led to an additional saving of ≈60 EUR∙m^−2^ during the 50 years;
- There was a steeper decrease in the annual energy consumption with thermal renders up to 0.02 m thickness in the Azores and 0.04 m in Bragança, leading to absolute energy savings of ≈5 and 15 kWh∙m^−2^∙year^−1^, respectively, when compared with the uninsulated wall;
- A thickness of 0.01 m of thermal render can lead to annual operational energy savings of 7.86 kWh∙m^−2^ in Bragança and of 2.10 kWh∙m^−2^ in the Azores;
- Optimum thickness related to the ADP-ff and GWP impacts are higher than concerning economic assessment, resulting in 0.04 m for the Azores and 0.09 m for Bragança;
- The optimised formulations led to economic and environmental savings, especially from 600 DD* onwards.

Thus, different geographical locations, application conditions and primary objectives define the most adequate thermal insulating material. Nonetheless, in this integrated study, TRopt sisal showed lower costs, higher energy savings and lower environmental impacts for the considered indicators. In summary, the results of this research can support designing innovative and multiperformance aerogel-based fibre-enhanced thermal render solutions for buildings’ facades since their application can potentially save money, energy and environmental impacts, providing building energy conservation and sustainability.

## 4. Recommendations

An opportunity exists for further work investigating other aspects of the application of the aerogel-based fibre-enhanced thermal renders, such as in retrofit scenarios, which represent the current most common use of aerogel in buildings [71], and in comparison with other thermally insulating materials as a benchmark. Moreover, future investigation paths concern dynamic energy simulations of the innovative thermal renders application on building facades while evaluating moisture influence on performance over time. The association of an environmental, economic and energy study with hygrothermal simulation models could lead to interesting results.

Other occupation periods of the constructions could be studied to evaluate the impact on energy needs. The materials’ durability should be assessed to verify their ability to accomplish the designed service life, possibly providing new insights for formulation development. If the primary objective of an optimisation method is to test thermal insulation regulations (usually translated into a maximum U-value), the constructive solution must be regarded as a whole.

## 5. Materials and Methods

### 5.1. Climate

Portugal is located in the Western part of Europe, under the influence of the Atlantic Ocean. According to the Köppen climate classification [72,73], most of Portugal’s mainland territory presents a temperate continental climate, Type *C*, with the subtype *Cs* (temperate climate with dry summer) and the varieties *Csa*, temperate climate with warm and dry summer, and *Csb*, temperate climate with dry and mild summer. Concerning the Portuguese islands, Madeira is type *Csa*, and the Azores Eastern Group and Central and West Group are *Cfb*, temperate maritime climate, representing the Mediterranean climate.

The number of degree-days is commonly used to estimate the energy required for cooling and heating [39,41,70]. Degree-days are divided into CDD and HDD and calculated based on the observation of long-term climatic data for any location compared with the indoor reference temperatures for the cooling and heating seasons. In Portugal, the reference indoor temperatures are 25 °C for the cooling and 18 °C for the heating season [64], with the cooling season presenting a four-month duration [74]. 

Equations (1) and (2) calculate the temperature differences in terms of the number of days when the mean outdoor temperature is above or below the reference temperature [6,70]. Equation (1) should be used when T_out_ ≥ T_in_ with T_in_ = 25 °C, in which T_out_ is the daily mean outdoor air temperature [°C] and T_in_ is the indoor reference air temperature [°C], and Equation (2) should be used when T_out_ ≤ T_in_ with T_in_ = 18 °C

(1) $$CDD=\sum_{days}\left(T_{out}-T_{in}\right)$$

(2) $$HDD=\sum_{days}\left(T_{in}-T_{out}\right)$$

where CDD is the cooling and HDD the heating degree-days [°C∙day].

In Portugal, the HDD are available in the legislation [74] for all the regions (Nomenclature of Territorial Units for Statistics—NUTS). The CDD values are calculated based on the air temperature available in meteorological stations [75,76], averaged for at least five years, using Equations (1) and (2).

As a simplification, the HDD and CDD were conjugated in a single parameter for graphical representation using Equation (3). Therefore, an equivalent degree-days was considered: DD* [6,51], in [°C∙day], defined as a function of the climate (HDD and CDD) and of the energy efficiency of the heating (COP) and cooling (EER) systems. With DD*, the climate findings may be translated to other climatic conditions.

(3) $${DD}^{*}=\left(\frac{HDD}{COP}+\frac{CDD}{EER}\right)$$

Table 2 synthesises the climatic conditions for several Portuguese cities.

In Table 2, the areas with the most differentiated conditions are the Azores (most temperate: fewer energy demands) and Bragança (coldest and with an intermediate need for cooling: higher energy demands). Therefore, these two locations were selected for further study since the conditions of the other regions are in between them.

### 5.2. Aerogel-Based Fibre-Enhanced Thermal Renders

#### 5.2.1. Characterisation and Selection of Formulations

A previously developed aerogel-based thermal render [9] was used, for which the partial substitution of the reference render powder by fibres led to mechanical, physical and environmental improvements [26]. The used fibres were from synthetic and natural origins: aramid, sisal and biomass, with the best performances obtained with 0.50%, 0.10% and 0.10% substitution quantities (by total volume), respectively.

The reference aerogel-based thermal render was composed of Portland cement and calcium aluminate cement (20% m/m), rheological and hydrophobic agents, resins and granules of supercritical silica aerogel (37% m/m), which occupied approximately 70% of the volume of the mixtures. The ratio of water to render powder was 1.3:1.0 (without considering the mass of fibres), and batches were produced with around 1400 mL [9,26].

The main parameters of the fibres are shown in Table 3 and have supported the selection of formulations to be further studied, looking for the highest characteristic variations between them. To visually identify the main differences among the formulations (TR aramid, TR sisal and TR biomass) and the reference thermal render (TR reference), a colour scheme was adopted in Table 3: green identifies a better performance than TR reference; orange, a worse performance; and no colour indicates a similar performance to TR reference or a result with no significant importance. The formulations showing the highest variation and which had the highest potential for further study were TR aramid and TR sisal, with the others showing intermediary behaviour.

The LCA environmental indicators investigated refer to an A1 to A3 scenario (cradle to gate). Maintenance and end-of-life stages were not considered as they were similar for all the solutions. The two critical environmental indicators studied were ADP-ff (abiotic depletion potential from fossil fuels) and GWP (global warming potential), as a measure of non-renewable energy and greenhouse gas emissions, respectively, whose results were based on a previous study [30].

Table 3 also shows the high cost of the formulations, which gave rise to a sensitivity analysis. As for the fibres, since they were bought in small quantities and, thus, did not reflect the accurate bulk prices, a verification of their current industrial prices was performed. The aramid fibres cost around 30,000 USD∙m^−3^ [77], being herein considered a cost conversion to 26,000 EUR∙m^−3^. The sisal fibres present market values of around 1600 USD∙ton^−1^ [78], corresponding to 1067 EUR∙m^−3^. Finally, due to their novelty and resulting from recent investigation works [79], the biomass fibres present a cost of 3842 EUR∙m^−3^, which can be significantly reduced with proper industrial development. The final costs represent the respective fibre volume substitution in each formulation.

#### 5.2.2. Sensitivity Analysis of the Silica Aerogel Cost

The production costs were obtained with the aerogel-based thermal render manufacturer, reflecting acquisition costs not negotiated with the raw materials producers. Thus, these formulations presented a high acquisition cost per cubic meter, which motivated a more detailed analysis to verify if there was potential for reductions. Although fibre use has economic impacts, even the TR reference showed high costs (2478 EUR∙m^−3^). Therefore, the other formulations represented even higher expenses.

**Figure 6:**
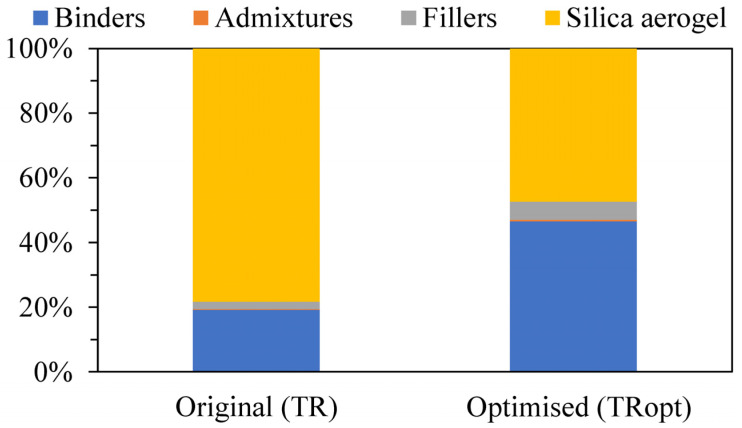
Original (TR) and optimised (TRopt) cost scenarios of the aerogel−based thermal render formulation per cubic meter.

An investigation was carried out to identify which raw materials impacted the TR reference costs the most (Figure 6: Original scenario (TR)). The binders represented ≈19% of the cost per cubic meter, with the silica aerogel representing more than 75% and the admixtures and fillers only ≈3%. Thus, silica aerogel was the raw material with the highest contribution to the renders’ acquisition cost, which is also due to the significant amount used in the formulations (≈70% vol.).

Although the silica aerogel costs are currently around 3000 EUR∙m^−3^, without negotiation and as reported by the render’s manufacturer, a significant potential for price reduction was found in Koebel et al. [14] and Cuce et al. [50] for the near future, hitting values as low as 600 EUR∙m^−3^ (or ≈6700 EUR∙ton^−1^, for a bulk density of ≈ 90 kg∙m^−3^ [80,81]), by optimising the synthesis processes [30,82,83].

Such a decrease in the silica aerogel cost enabled an optimised cost scenario (Figure 6: Optimised scenario (TRopt)). The binders’ contribution increased to ≈45% of the cost per cubic meter, while the silica aerogel dropped to ≈45%, with the admixtures and fillers representing only ≈10%. Therefore, the optimised cost scenario lowered the original TR reference cost from 2478 EUR∙m^−3^ to 1021 EUR∙m^−3^, corresponding to a ≈60% cost reduction. Thus, the acquisition costs of TR aramid and TR sisal were divided into two economic scenarios: non-optimised and optimised. For the TR aramid, the non-optimised cost was 2596 EUR∙m^−3^, while the optimised was 1146 EUR∙m^−3^. For the TR sisal, the non-optimised cost was 2477 EUR∙m^−3^, and the optimised was 1021 EUR∙m^−3^.

#### 5.2.3. Description of the Walls

**Figure 7:**
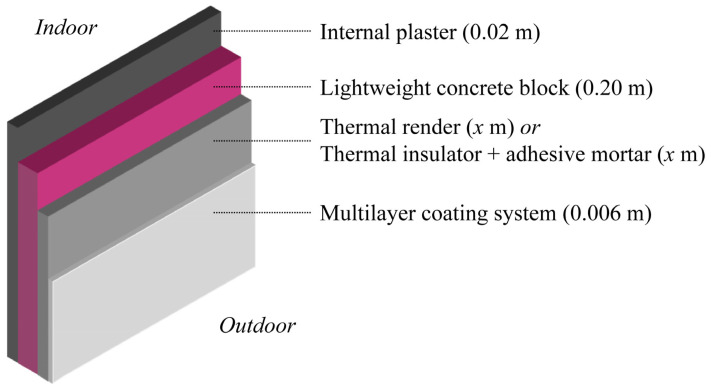
Single−leaf wall composition.

**Figure 8:**
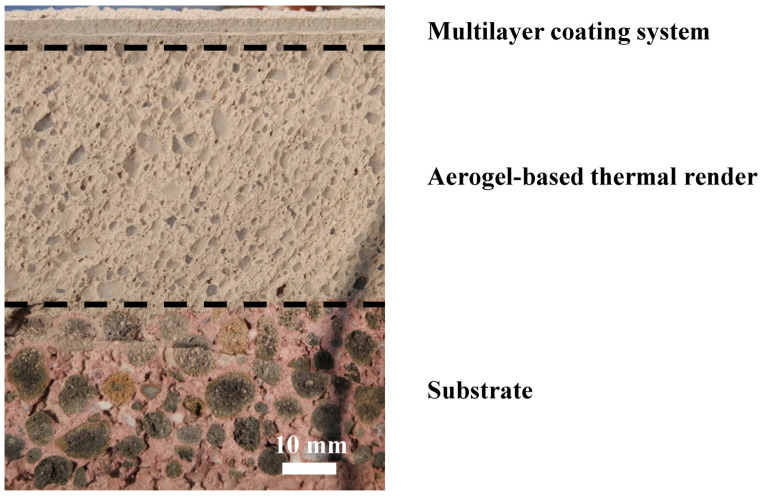
Section of the aerogel-based thermal render applied on-site over a lightweight concrete block.

A single-leaf composition was chosen to study the impact of aerogel-based fibre-enhanced thermal renders on exterior walls, reflecting new construction practices (Figure 7). Considering that the substrate (lightweight concrete block) is adequately flat, the thermal renders can be directly applied on its surface (Figure 7 and Figure 8) [27], with a maximum thickness of 0.08 m, as prescribed by the manufacturers. Figure 8 shows the aerogel-based thermal render solution applied in a real scenario.

In the wall, the external finishing multilayer coating system, the internal plaster and the lightweight concrete blocks were similar in all simulations, and their thermal transmittance (U) contribution was considered. The multilayer coating system comprises commercially available basecoat, fibreglass mesh, keycoat and acrylic finishing coat, with a total thickness of ≈0.006 m, previously characterised in terms of environmental impacts [84].

### 5.3. Methodology—Numerical Simulation Model

Numerical simulations were performed to promote an integrated analysis, considering the economic, energy and environmental aspects, and the thermal insulation thickness optimisation. The calculation of the optimum insulation thicknesses [37,85,86] was carried out. In this approach, as the thermal insulation thickness increases, its embodied cost and environmental impacts also increase. In contrast, the heating and cooling energy needs and, consequently, the operational cost and following environmental impacts decrease. Therefore, the optimum thermal insulation thickness has a minimal total cost, environmental impacts and energy consumption over the building service life.

Different scenarios were evaluated with the numerical simulation. The energy savings were reflected in the economic and environmental impacts considering aramid and sisal-containing formulations compared without economic optimisation (TR aramid and TR sisal) and with the optimisation in costs (TRopt aramid and TRopt sisal). The aim was to evaluate the implications and performance differences between economically non-optimised and optimised formulations when applied to a new wall, based on the constructive solution presented in Figure 7, without any direct concerns about legislative requirements related to the walls’ thermal transmittance (U). The simulations were performed from 0 to 0.10 m of thermal insulation layer thickness in 0.01 m increments.

#### 5.3.1. Materials and Processes Input

Table 4 presents the characteristics of the materials used in the simulations. For the environmental characterisation, it was considered the most similar processes available in the *Ecoinvent 3* database (v3.4) [87] present in the *SimaPro* software (v8.5.2.0). When available, the values were compared with the results in the literature (Table 5).

Since the use of thermal insulation materials impacts the heating and cooling energy needs of buildings, and, in Portugal, the indoor temperature is usually regulated by electrical equipment [31,90], the Portuguese environmental, energy and economic characteristics of electricity mix are presented in Table 6.

#### 5.3.2. Calculation Parameters Input

A set of parameters shown in Table 7 was considered in the numerical model, representing geographical location influence, energy options, materials and economic factors.

The indoor air film thermal resistance (R_in_ [m^2^∙K∙W^−1^]), the outdoor air film thermal resistance (R_out_ [m^2^∙K∙W^−1^]), the energy efficiency ratio of the heating system (COP) and the energy efficiency ratio of the cooling system (EER), were based on the Portuguese legislation [92,93]. For the interest and inflation rates, without considering risk, data available from the Portuguese National Statistics Institute (INE) [79] were used. The thermal insulation service life was considered to correspond to the building’s expected service life of 50 years. The impacts of maintenance were considered similar, and, thus, this was not taken into account.

#### 5.3.3. Heating and Cooling Energy Consumption

Equation (4) [94] was used to evaluate an external wall’s thermal loss per unit area.

(4) $$q=U(T_{in}-T_{out})$$

where q is the heat loss per unit area [W∙m^−2^], U is the thermal transmittance [W∙m^−2^∙°C^−1^], T_in_ is the constant indoor air temperature [°C] and T_out_ the mean daily outdoor air temperature [°C].

The U-value is calculated for the wall using Equation (5) [94], given that the thermal conductivity (λ) [W∙m^−1^∙K^−1^] and the thickness (x) [m] is known for each material, allowing us to obtain their respective thermal resistances (R) [m^2^∙K∙W^−1^] using Equation (6) [94].

(5) $$U=\frac{1}{R_{in}+R_{w}+R_{ins}+R_{out}}$$

(6) $$R=\frac{\lambda }{x}$$

where R_in_ is the indoor air film thermal resistance [m^2^∙K∙W^−1^], R_w_ is the wall thermal resistance (without thermal insulation) [m^2^∙K∙W^−1^], R_ins_ is the thermal resistance of the insulation material [m^2^∙K∙W^−1^] and R_out_ is the outdoor air film thermal resistance [m^2^∙K∙W^−1^].

It is then possible to evaluate the annual energy requirements for heating (E_heat_) and cooling (E_cool_) per unit area of an external wall [J∙m^−2^∙year^−1^] using Equations (7) and (8) [86], respectively.

(7) $$E_{heat}=\frac{86,400\cdot U\cdot HDD}{COP}$$

(8) $$E_{cool}=\frac{86,400\cdot U\cdot CDD}{EER}$$

where COP and EER are the energy efficiency ratios of the heating and cooling systems, respectively, and HDD and CDD are the heating and cooling degree-days, respectively [°C∙day].

The annual energy consumptions for heating (E_cons,heat_) and cooling (E_cons,cool_) [kW∙h∙m^−2^] were calculated using Equations (9) and (10), respectively [39,43].

(9) $$E_{cons,heat}=\frac{86,400\cdot U\cdot HDD}{COP\cdot H_{u}}$$

(10) $$E_{cons,cool}=\frac{86,400\cdot U\cdot CDD}{EER\cdot H_{u}}$$

where H_u_ is the lower heating value of the used energy source (3.60 × 10^6^ [J∙kW∙h^−1^], for electricity).

The sum of the heating and cooling consumptions represented the total annual energy required.

#### 5.3.4. Economic Analysis

Based on the calculations of the annual consumption for heating and cooling, as well as on the energy source cost (C_e_) [EUR∙kW^−1^∙h^−1^] for electricity, the yearly energy use costs per surface unit area (C_use_) [EUR∙m^−2^∙year^−1^] were estimated. This includes the needs of heating and cooling seasons (Equation (11) [39,40,85,86]) satisfied by a piece of electric equipment.

(11) $$C_{use}=\left(\frac{86,400\cdot U\cdot HDD}{COP\cdot H_{u}}+\frac{86,400\cdot U\cdot CDD}{EER\cdot H_{u}}\right)\cdot C_{e}$$

The cost associated with the thermal insulation (C_ins_) [EUR∙m^−2^], per unit area, was calculated with Equation (12).

(12) $$C_{ins}=C_{i}\cdot x$$

where C_i_ corresponds to the price of the thermal insulation material [EUR∙m^−3^] and x to the thermal insulation thickness [m].

Since C_use_ only considers the costs over one year and the service life is five decades, the PWF (present worth factor) method [37,39,40,43,46,70] was applied to optimise the energy cost. Therefore, the cost of the needed energy as a function of the wall’s thermal insulation thickness was calculated considering the service life of the thermal insulation (N) [year], the interest rate (i) and the inflation rate for the energy cost (g), without considering any risk. Therefore, the cash flows were converted to a single equivalent sum at time zero [95]. Due to economic variations, the interest rate (i) was adjusted for the inflation rate, designated as the actual interest rate (r) [95], which affects the calculation of the PWF (Equation (13)) [46,86].

(13) $$PWF=\frac{{\left(1+r\right)}^{N}-1}{r\cdot {\left(1+r\right)}^{N}},\left\{\begin{array}{c} i>g:\;\;r=\frac{i-g}{1+g}\\ i<g:\;\;r=\frac{g-i}{1+i} \end{array}\right.$$

If i = g, the PWF is calculated using Equation (14) [46,86].

(14) $$PWF=\frac{N}{1+i}$$

Equation (11) was then updated to represent the energy use costs during the considered period (C_use,SL_) [EUR∙m^−2^] (Equation (15)).

(15) $$C_{use,SL}=\left(\frac{86,400\cdot U\cdot HDD}{COP\cdot H_{u}}+\frac{86,400\cdot U\cdot CDD}{EER\cdot H_{u}}\right)\cdot C_{e}\cdot PWF$$

The total cost for the service life of each solution (C_total,SL_) [EUR∙m^−2^] can be evaluated considering both the thermal insulation’s acquisition cost and the energy consumption’s present value (Equation (16)) [40,85].

(16) $$C_{total,SL}=\left(\frac{86,400\cdot U\cdot HDD}{COP\cdot H_{u}}+\frac{86,400\cdot U\cdot CDD}{EER\cdot H_{u}}\right)\cdot C_{e}\cdot PWF+C_{ins}$$

The savings during the solution’s service life (S_SL_) [EUR∙m^−2^] were calculated by the cost difference between the uninsulated and the insulated wall through time (Equation (17)) [6].

(17) $$S_{SL}=\left(\frac{86,400\cdot \left(U_{un}-U_{ins}\right)\cdot HDD}{COP\cdot H_{u}}+\frac{86,400\cdot \left(U_{un}-U_{ins}\right)\cdot CDD}{EER\cdot H_{u}}\right)\cdot C_{e}\cdot PWF-C_{ins}$$

where U_un_ and U_in_ correspond to the thermal transmittance [W∙m^−2^∙°C^−1^] of the wall thermally uninsulated and insulated, respectively.

Equation (15) is minimised to obtain the optimum thickness of thermal insulation considering the costs involved: the derivative for x (thickness) is taken and set equal to zero (since C_ins_ is calculated from the product between C_i_ and x), from which the optimised thickness (x_opt_) is obtained using Equation (18) [38,43,86]. R_wt_ is the sum of R_in_, R_w_ and R_out_.

(18) $$x_{opt}=293.94\cdot {\left(\frac{HDD\cdot C_{e}\cdot PWF\cdot \lambda_{ins}}{COP\cdot H_{u}\cdot C_{i}}+\frac{CDD\cdot C_{e}\cdot PWF\cdot \lambda_{ins}}{EER\cdot H_{u}\cdot C_{i}}\right)}^{\frac{1}{2}}-R_{wt}\cdot \lambda_{ins}$$

Equation (19) calculates the payback period (PP) [year] related to the application of thermal insulation materials [40,86]. It divides the thermal insulation cost (C_ins_) by the annual energy cost savings (S_ES_) [EUR∙m^−2^∙year^−1^] since similar energy consumption is considered during each solution service life. S_ES_ is calculated by subtracting the thermally insulated wall’s annual costs from those of the non-insulated wall [39,41,50].

(19) $$PP=\frac{C_{ins}}{S_{ES}}$$

#### 5.3.5. Environmental Analysis

A constant electricity mix was assumed during the service life for the environmental evaluation. Knowing the ADP-ff and the GWP impacts of the electricity consumption per kWh (Table 6) and the impacts from the thermal insulation materials (Table 4), it was possible to estimate the impacts for both environmental indicators. The impacts of the electricity per unit area were calculated based on the sum of the E_cons,heat_ and E_cons,cool_, which give the total annual kWh∙m^−2^, multiplied by the respective environmental impact attributed to the electricity mix per kWh (Table 6).

Similarly, to the thermal insulating costs, the environmental impact of the thermal insulating materials was evaluated by multiplying their thickness, x [m], by the environmental impact for each cubic meter of material (Table 4). A similar calculation as in [39,96] was followed to analyse the environmental payback of the solutions (PP_ADP-ff OR GWP_) [year] (Equation (20)) [39,96]. The calculation computed the impacts of the considered service life (50 years).

(20) $${PP}_{ADP-ff\;OR\;GWP}=\frac{I_{ins}}{I_{sol\;without\;ins}-I_{sol\;with\;ins}}$$

where I_ins_ is the impact of the thermal insulation material for a given thickness, I_sol without ins_ corresponds to the impacts of the energy consumption in an uninsulated wall and I_sol with ins_ to the impacts of the energy consumption in an insulated wall. The units of ADP-ff and GWP are [MJ∙m^−2^∙year^−1^] and [kg CO_2_ eq∙m^−2^∙year^−1^], respectively.

The annual impacts were multiplied by the number of years of the service life to select the optimised environmental impact thickness (corresponding to the lowest value of the sum of the impacts throughout the service life).

## Figures and Tables

**Table 1:** Optimum insulation thickness, costs and environmental impact savings for a new wall.

Region (DD*)	Render Designation	U [W∙m^−2^∙°C^−1^]	x_opt_ [m]	C_ins_ [EUR∙m^−2^]	S_SL_ [EUR∙m^−2^]	PP [Year]	SL ADP-ff Savings [MJ∙m^−2^]	PP ADP-ff [Year]	SL GWP Savings [kgCO_2_ eq∙m^−2^]	PP GWP [Year]
Azores (188.8)	TR aramid	0.99	0.01	25.96	0.05	>50	313.80	11.4	36.61	8.2
	TR sisal	0.98	0.01	24.77	2.42	>50	340.78	9.9	39.08	7.3
	TRopt aramid	0.75	0.02	22.93	16.60	>50	432.20	15.1	52.17	10.8
	TRopt sisal	0.73	0.02	20.43	20.45	49.8	470.31	13.2	55.41	9.8
Bragança (680.1)	TR aramid	0.61	0.03	77.88	94.41	41.2	2415.23	5.2	268.56	3.7
	TR sisal	0.59	0.03	74.32	102.67	36.1	2514.39	4.6	277.92	3.4
	TRopt aramid	0.44	0.05	57.33	149.65	19.1	2771.74	7.2	312.57	5.2
	TRopt sisal	0.36	0.06	61.30	160.42	19.1	2959.73	7.3	333.03	5.4

Note: U—thermal transmittance [W∙m^−2^∙°C^−1^]; x_opt_—optimum thickness [m]; C_ins_—insulation cost [EUR∙m^−2^]; S_SL_—savings during the considered service life [EUR∙m^−2^]; PP—payback period [year]; SL ADP-ff savings—ADP-ff savings during the considered service life [MJ∙m^−2^]; SL GWP savings—GWP savings during the considered service life [kgCO_2_ eq∙m^−2^].

**Table 2:** Climatic data for some Portuguese cities.

Location	City	Alt. [m] [76]	Long. [°] [76]	Lat. [°] [76]	Köppen Class [76]	HDD [°C∙Day] Base 18 °C [74]	CDD [°C∙Day] Base 25 °C [64,75]
Islands	**Azores—Ponta Delgada**	**48**	**25W40**	**37N43**	**Cfb**	**604**	**0**
	Madeira—Funchal	380	16W54	32N37	Csa	618	18
Mainland	Faro	9	7W55	37N1	Csa	987	98
	Lisboa	4	9W7	38N43	Csa	1071	105
	Évora	240	7W54	38N34	Csa	1150	289
	Porto	94	8W36	41N8	Csb	1250	27
	Castelo Branco	348	7W29	39N49	Csa	1274	278
	Coimbra	151	8W25	40N12	Csb	1304	32
	Vila Real	450	7W44	41N18	Csb	1764	121
	Guarda	1056	7W15	40N32	Csb	1924	16
	**Bragança**	**674**	**6W45**	**41N48**	**Csb**	**2015**	**136**

Note: **bold**—locations selected for simulation; Parameters: Alt.—altitude [m]; Long.—longitude [degree]; Lat.—latitude [degree]; HDD—heating degree-days [°C∙day]; CDD—cooling degree-days [°C∙day].

**Table 3:** Performance of the aerogel-based fibre-enhanced thermal render formulations.

Group of Tests	Parameter	Render Designation			
		TR Reference	TR Aramid	TR Sisal	TR Biomass
Fresh state [26]	Water powder ratio	1.3	1.3	1.3	1.3
	Workability	Excellent	Good	Excellent	Excellent
	Consistency [mm]	143.5	121.1	139.7	139.1
	Bulk density [kg∙m^−3^]	293	310	297	299
	Air content [%]	21.5	19.5	22	22.5
Mechanical [26]	Bulk density [kg∙m^−3^]	159	165	160	162
	Compressive strength, peak [MPa]	0.185	0.208	0.193	0.19
	Flexural strength, peak [MPa]	0.092	0.165	0.093	0.092
	Cracks during curing (visual evaluation)	No	No	No	No
	Dynamic modulus of elasticity [MPa]	51.3	77.4	49.2	48.3
Impact [26]	Adhesive strength to block [MPa]	0.066:B	0.075:B	0.067:B	0.066:B
	Adhesive strength to basecoat [MPa]	0.065:B	0.073:B	0.065:B	0.066:B
	Diameter impact 3 J [mm]	31.5	29.9	31	31.2
	Cracks (number of impacts with cracks in 5 impacts)	3 in 5	0 in 5	1 in 5	2 in 5
	Pendulum hammer index	59.3	62.4	59.5	59.4
Physical [26,29]	Water absorption coefficient [kg∙m^−2^∙s^−1/2^]	0.109	0.0286	0.0325	0.031
	Open porosity [%]	86.3	85.1	86.9	87
	Thermal conductivity at 10 °C and dry-state [W∙m^−1^∙K^−1^]	0.029	0.032	0.030	0.031
	Thermal conductivity at 10 °C and saturated [W∙m^−1^∙K^−1^]	0.1401	0.1311	0.123	0.1285
	Water content at 80%RH [kg∙m^−3^]	7.8	7.12	7.56	7.28
	Water content saturation by capillary action [kg∙m^−3^]	281.04	246.08	260.2	274.82
	Water vapour diffusion resistance factor	13.7	13.3	12.7	12.4
LCA [30]	ADP-ff [MJ], per cubic meter	8494.9	9303.2	8452.3	8459.3
	GWP [kg CO_2_ eq], per cubic meter	675.1	720.4	672.2	672.8
Economic	Cost [EUR∙m^−3^]	2478.76	2596.37	2477.35	2480.12

Legend: 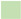
—Improved performance over reference; 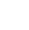
—Similar performance to reference or non-significant; 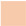
—Worse performance over reference.

**Table 4:** Properties of the materials.

Material	Density [kg∙m^−3^]	Thickness [m]	Λ [W∙m^−1^∙K^−1^]	R [m^2^∙K∙W^−1^]	Cost [EUR∙m^−3^]	ADP-ff [MJ∙m^−3^]	GWP [kg CO_2_ eq∙m^−3^]
Internal plaster [88]		0.020		0.011			
Lightweight concrete block [88]		0.200		0.520			
Multilayer coating system [27]		0.0055		0.007			
TR cork [89]	825		0.095		566.0 *	2739.0	333.3
TR aramid	165		0.032		2596.3	9303.2	720.4
TR sisal	160		0.030		2477.3	8452.3	672.2
TRopt aramid	165		0.032		1146.5	9303.2	720.4
TRopt sisal	160		0.030		1021.6	8452.3	672.2

Note: * prices obtained by averaging values of several distributors; TR cork—industrial thermal render with cork granules; TR aramid—aerogel-based thermal render with 0.50% *v/v* of aramid fibres; TR sisal—aerogel-based thermal render with 0.10% *v/v* of sisal fibres; TRopt aramid—aerogel-based thermal render with 0.50% *v/v* of aramid fibres with optimised aerogel economic costs; TRopt sisal—aerogel-based thermal render with 0.50% *v/v* of sisal fibres with optimised aerogel economic costs.

**Table 5:** Processes and references used for the environmental characterisation of the thermal insulation materials.

Material	Ecoinvent Process and Literature References
TR cork	[32]
TR aramid	[84]
TR sisal	[84]

**Table 6:** Environmental, energy and economic characteristics of the electricity mix used in Portugal [87,91].

Ecoinvent Process	Cost [EUR∙kWh^−1^]	ADP-ff [MJ∙kWh^−1^]	GWP [kg CO_2_ eq∙kWh^−1^]	Lower Heating Value (H_u_) [J∙kW∙h^−1^]
Electricity, low voltage {PT}|market for|Cut-off, S	0.22	3.90	0.42	3.60 × 10^6^

**Table 7:** Calculation parameters.

Parameter	Value
Locations	Table 2
CDD [°C∙day]	Table 2
HDD [°C∙day]	Table 2
TR/TRopt aramid and sisal	Table 4
Other materials’ characteristics	Table 4
R_in_ [m²∙K∙W^−1^]	0.13 [92]
R_out_ [m²∙K∙W^−1^]	0.04 [92]
Electricity	Table 6
COP (multisplit)	3.40 [93]
EER (multisplit)	3.00 [93]
Interest rate (i)	3.0% [79]
Inflation rate (g)	2.0% [79]
Service life new wall, N_new_ [year]	50

Note: HDD—heating degree-days [°C∙day]; CDD—cooling degree-days [°C∙day]; TR aramid—aerogel-based fibre-enhanced thermal render with 0.50% *v/v* of aramid fibres; TR sisal—aerogel-based fibre-enhanced thermal render with 0.10% *v/v* of sisal fibres; R_in_—indoor air film thermal resistance [m^2^∙K∙W^−1^]; R_out_—outdoor air film thermal resistance [m^2^∙K∙W^−1^]; COP—energy efficiency ratio of the heating system; EER—energy efficiency ratio of the cooling system; i—interest rate [%]; g—inflation rate [%]; N_new_—service life of the new wall [year].

## Data Availability

The data presented in this study are openly available in article.

## Abbreviations

Abbreviation	Meaning
ADP-ff	Abiotic depletion potential from fossil fuels [MJ]
Az	The Azores
Br	Bragança
CDD	Cooling degree-days [°C∙day]
COP	Energy efficiency ratio of the heating system
C_e_	Energy cost [EUR∙kW^−1^∙h^−1^]
C_i_	Thermal insulating material cost [EUR∙m^−3^]
C_ins_	Thermal insulating material cost per unit area [EUR∙m^−2^]
C_use_	Annual costs of energy per surface area [EUR∙m^−2^]
C_use,SL_	Service life costs of energy per surface area [EUR∙m^−2^]
C_total,SL_	Service life costs of the solution per surface area [EUR∙m^−2^]
DD*	Degree-days as a fraction of climate and energy efficiency [°C∙day]
EER	Energy efficiency ratio of the cooling system
EPS	Expanded polystyrene
ETICS	External thermal insulation composite systems
E_cons,heat_	Annual energy consumption for heating [kWh∙m^−2^]
E_cons,cool_	Annual energy consumption for cooling [kWh∙m^−2^]
E_cool_	Annual energy requirements for cooling [J∙m^−2^]
E_heat_	Annual energy requirements for heating [J∙m^−2^]
GWP	Global warming potential [kg CO_2_ eq]
g	Inflation rate
HDD	Heating degree-days [°C∙day]
H_u_	Lower heating value [J∙kW^−1^∙h^−1^]
i	Interest rate
N	Lifetime [year]
PP	Payback period [year]
PWF	Present worth factor
q	Heat loss per unit area [W∙m^−2^]
R	Thermal resistance [m^2^∙K∙W^−1^]
R_in_	Indoor air film thermal resistance [m^2^∙K∙W^−1^]
R_ins_	Thermal insulation material thermal resistance [m^2^∙K∙W^−1^]
R_out_	Outdoor air film thermal resistance [m^2^∙K∙W^−1^]
R_w_	Wall thermal resistance [m^2^∙K∙W^−1^]
r	Interest rate adjusted for the inflation rate
S_ES_	Annual energy cost savings [EUR∙m^−2^∙year^−1^]
S_SL_	Savings achieved during the service life [EUR∙m^−2^]
TR	Thermal render
TRopt	Cost-optimised thermal render
TR cork	Thermal render with cork granules
T_in_	Mean daily indoor temperature [°C]
T_out_	Mean daily outdoor temperature [°C]
U	Thermal transmittance [W∙m^−2^∙°C^−1^]
U_in_	Thermal transmittance of an insulated wall [W∙m^−2^∙°C^−1^]
U_un_	Thermal transmittance of an uninsulated wall [W∙m^−2^∙°C^−1^]
x	Thickness [m]
x_opt_	Optimised thickness [m]
λ	Thermal conductivity [W∙m^−1^∙K^−1^]
